# Metastasis-Associated Protein 1 Is Involved in Angiogenesis after Transarterial Chemoembolization Treatment

**DOI:** 10.1155/2017/6757898

**Published:** 2017-05-15

**Authors:** Tao Xue, Wenming Feng, Hongbin Yu, Ming Zhu, Maoyun Fei, Ying Bao, Xiaoyi Wang, Wenxue Ma, Guiyuan Lv, Jianming Guan, Suhong Chen

**Affiliations:** ^1^College of Pharmaceutical Sciences, Zhejiang Chinese Medical University, Hangzhou 310053, China; ^2^Laboratory of Molecular Medicine, First People's Hospital Affiliated to Huzhou University, Huzhou 313000, China; ^3^Department of Hepatobiliary Surgery, First People's Hospital Affiliated to Huzhou University, Huzhou 313000, China; ^4^Department of Nephrology, First People's Hospital Affiliated to Huzhou University, Huzhou 313000, China; ^5^Moores Cancer Center, University of California, San Diego, CA 92037, USA; ^6^Department of Ultrasound, First People's Hospital Affiliated to Huzhou University, Huzhou 313000, China; ^7^Collaborative Innovation Center of Yangtze River Delta Region Green Pharmaceuticals, Zhejiang University of Technology, Hangzhou 310014, China

## Abstract

**Background:**

Transarterial chemoembolization (TACE), a well-established treatment for unresectable hepatocellular carcinoma (HCC), blocks the arterial blood supply to the tumor, which can be short-lived as development of collateral neovessels, leading to the failure of treatment. Metastasis-associated protein 1 (MTA1) is involved in development of tumors and metastases. However, the role of MTA1 in angiogenesis is still obscure.

**Methods:**

We detected the expression of MTA1 and hypoxia-inducible factor-1*α* (HIF-1*α*) and microvessel density (MVD) value in liver tumor tissues and tumor periphery before and after TACE treatment. Hepatocellular carcinoma cell line HepG2, tube formation assay, and chorioallantoic membrane (CAM) assay were applied to explore the mechanism of MTA1 in angiogenesis.

**Results:**

We found that expression of MTA1 increased after TACE treatment, especially in tumor periphery, which was accompanied by markedly elevated MVD value, indicating a significant correlation between MTA1 and MVD value. Moreover, MTA1 contributed to neovascularization of residual tumors. Cellular experiments further revealed that MTA1 increased the stability and the expression of HIF-1*α*, and overexpression of MTA1 enhanced tube formation and neovessels of chick embryos.

**Conclusions:**

MTA1 is an active angiogenic regulator; our results shed light on better understanding in neovascularization, which are helpful to predict prognosis of TACE, and provide evidences for intervention to improve therapeutic effects on HCC.

## 1. Introduction

Hepatocellular carcinoma (HCC), a common malignant tumor, is the fifth leading cause of tumor death in the world with an estimated incidence of more than one million new cases per year [1]. The liver has the dual blood supply by the hepatic artery and portal vein [2]. Liver tumors derive their blood supply (90–95%) mainly from the hepatic artery, whereas the portal vein is responsible for about 80% of the blood supply to normal liver tissues [3, 4]. Therefore, transcatheter arterial chemoembolization (TACE), targeting necrosis and shrinkage of liver tumors, has been considered to be a promising treatment for HCC patients who are not suitable for surgical operation. However, the long-term efficacy of this treatment is not satisfactory; five-year survival rate is lower than 10% [5].

It is generally accepted that TACE obstructs arterial blood supply to liver tumors, which results in acute oxygen depletion and ischemia, and enhances hypoxia of tumor and hepatocytes [6]. Thus, TACE is expected to induce an environment unfavorable for cell growth. However, tumor cells exposed to hypoxia undergo a series of metabolic alterations that allow them to survive and even proliferate. The reasons underlying TACE treatment failures have been linked to neovascularization [7].

MTA1, the founding member of the MTA family, a component of the nucleosome remodeling and histone deacetylation (NuRD) complex [8], exhibits effects on contributing to the DNA damage response, controlling the steady state of proteins via protein ubiquitination, and maintaining expressed states in a wide variety of human epithelial malignancies [9, 10]. Recently, it is indicated that elevated expression levels of MTA1 in several tumor types, such as gastric, breast, colorectal, and esophageal carcinomas, appear to increase cell motility, potentiate growth, and enhance metastases [11, 12]. Therefore, MTA1 is an indicator of the potential aggressiveness of various tumors. Angiogenesis is also considered as a critical feature of invasion process. However, involvement of MTA1 in angiogenesis under TACE-caused hypoxia conditions has not been investigated so far, and the mechanism is not clear. Hypoxia-inducible factor-1 (HIF-1), composed of the oxygen-regulated HIF-1*α* subunit and the constitutively expressed HIF-1*β* subunit [13], is a key transcription factor that mediates response to hypoxia [14]; whether MTA1 interacts with HIF-1 remains obscure.

In the present study, we investigated the changes of MTA1 expression and angiogenesis in liver specimens, as well as the correlation between MTA1 and MVD value under hypoxia conditions after TACE. Hepatocellular carcinoma cell line HepG2, tube formation assay, and CAM assay were also applied to explore the preliminary mechanism of MTA1 in angiogenesis.

## 2. Materials and Methods

### 2.1. Patients

The diagnosis of HCC was based on European Association for the Study of Liver Disease [15] and confirmed by liver pathological histology. Patients were excluded if they had Child's class C liver dysfunction, eastern cooperative oncology group (ECOG) performance status > 2, extrahepatic disease, or thrombosis of the main portal vein. All procedures performed in the studies were in accordance with Helsinki declaration. The ethics committee approved the protocol (Application for Approval of Research Protocol number 2008-02), and informed consent was obtained from all patients. Finally, sixty-three patients with diagnosed unresectable hepatocellular carcinoma fulfilled the entry criteria in the study between January 2008 and Match 2014. The detailed and important characteristics of patients were shown in Table 1.

### 2.2. TACE Treatment

TACE was performed using gemcitabine 1000 mg/m^2^ (emulsified with Lipiodol, 10–12 mL), cisplatin 40 mg/m^2^, and 5-fluorouracil 500 mg/m^2^ with Embosphere® microspheres (Biosphere Medical, Rockland, USA). Chemotherapy and the embolic agents were administered through the hepatic artery to segmental and/or subsegmental feeding branches of the tumor. Stump occlusion of segmental or subsegmental feeding branches was performed with microfibrillar collagen (Avitene, Davol, Inc., Cranston RI, USA) as needed to achieve stasis. TACE was repeated on demand if recurrence was detected, and residual viable tumor tissue was evident without deterioration of hepatic function.

### 2.3. Ultrasound-Guided Liver Biopsy

**Figure 1:**
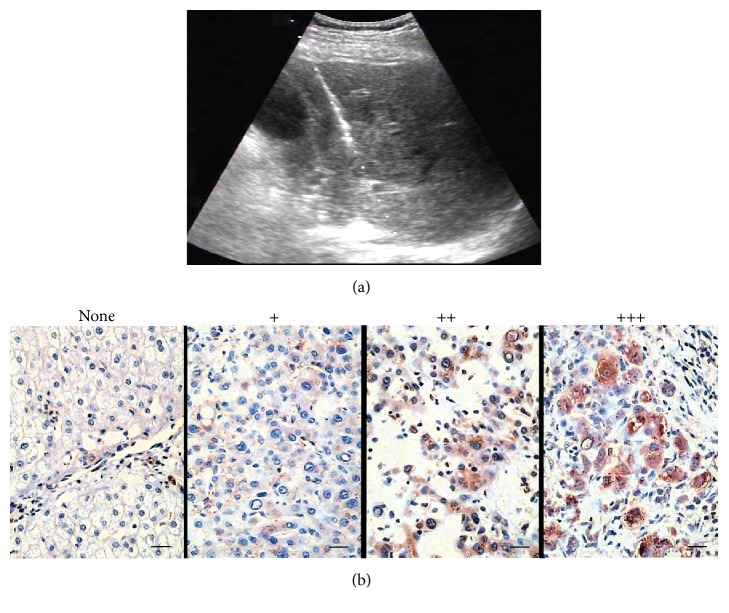
Ultrasound-guided liver biopsy and grade of immunohistochemical staining. (a) The percutaneous liver biopsies were performed by a radiologist using real-time ultrasound guidance. (b) Intensity of immunohistochemical staining was graded as none, +, ++, and +++, respectively (magnification = ×400, scale bar = 25 *μ*m).

The procedures were performed a subcostal or epigastric approach under local anesthesia in the intervention room. All patients underwent percutaneous ultrasound-guided core needle biopsies of the targeted liver tumors using 18-gauge needles (Bard Biopsy, Arizona, USA). The biopsy needle had a throw of 22 mm length to obtain biopsy samples from tumor tissues and tumor periphery (Figure 1(a)); ultrasound visualisation of the needle during the entire procedure was continuously maintained. Biopsies were carried out one week before TACE and one month and two months after TACE. The adequacy of the obtained tissues were immediately collected in 10% phosphate-buffered formalin and stored at −80°C, respectively.

### 2.4. Immunohistochemistry Analysis

Immunohistochemical staining was performed by avidin-biotin-peroxidase complex method and evaluated by two senior pathologists in a blinded manner. MTA1 and HIF-1*α* staining were recorded by a semiquantitative and grading system, as described previously for other markers [16]. Intensity was recorded as 0 (no staining) to 3 (strong staining), whereas the percentage of nuclear staining was recorded as 0 (no cells positive), 1 (positive staining in <10% of the cells), 2 (positive staining in 10–50% of the cells), 3 (positive staining in 51–75% of the cells), and 4 (positive staining in >75% of the cells). A staining index was calculated as the product of staining intensity and staining area. The cells were categorized as negative expressing (−), staining index 0; weakly positive expressing (+), staining indexes 1–4; moderately positive expressing (++), staining indexes 5–8; and strongly positive expressing (+++), staining indexes 9–12.

MVD was evaluated by means of the counting method based on Weidner criterion [17]. CD34-stained endothelial cell was counted as a single countable microvessel. The final MVD was the mean value obtained from the counts of the five fields.

### 2.5. Cell Culture and Preparation of Conditioned Media (CM)

HepG2 cells were maintained in DMEM containing 10% FBS. Hypoxic conditions (1% O_2_) were achieved by putting cells in a hypoxia incubator filled with a mixture of 1% O_2_, 5% CO_2_, and 94% N_2_. To generate mock and stable MTA1 transfectants, HepG2 cells were transfected with empty pcDNA3 or pcDNA3-Flag-MTA1. HepG2 mock or stable MTA1 transfectants were seeded and incubated for 24 h, and then culture supernatant was collected as conditioned media (CM).

### 2.6. Transient Transfection

pcDNA3-Flag-MTA1 plasmid and Gal4-HA-HIF-1*α* plasmid were purchased from Promega (Madison, WI, USA). Briefly, cells were transfected with indicated plasmids using Lipofectamine 2000 (Invitrogen, CA, USA), for 4 h in serum-free incubation, and then the culture medium was altered. After additional 20 h normal condition incubation, the cells were used in the following experiments.

### 2.7. Western Blot Analysis, Reverse Transcription PCR, Immunoprecipitation, and GST Pull-Down Assay

Extraction of the total cell or nuclear lysates and western blot analysis were performed as reported previously [18]. Reverse transcription PCR using published primer sequences and immunoprecipitation were also performed as described previously [19]. [^35^S]Methionine-labeled in vitro translated HIF-1*α* was prepared using the TNT system (Promega, WI, USA). GST-fusion proteins were expressed in* E. coli* BL21 (DE3) and induced with 0.4 mM isopropyl-thio-*β*-D-galactopyranoside. Equal amounts (1 *μ*g) of GST and GST-MTA1 immobilized on glutathione sepharose beads were incubated with in vitro translated HIF-1*α* in modified GBT buffer (10% glycerol, 50 mM Hepes-NaOH [pH 7.5], 170 mM KCl, 7.5 mM MgCl_2_, 0.1 mM EDTA, 0.1 mM DTT, and 1% Triton X-100) at 4°C for 3 h. After washing, the bound proteins were eluted with the sample buffer and were separated by SDS-PAGE, followed by western blot analysis.

### 2.8. Tube Formation Assay

Matrigel (10 mg/mL) was polymerized for 30 min at 37°C. HUVECs (2 × 10^4^) were seeded on the matrigel and grown in CMs. After 24 h of incubation, formation of tube-like structures was monitored by microscopic observation, and tube length was quantified using Image Lab imaging software.

#### 2.8.1. Chorioallantoic Membrane (CAM) Assay

The sterile filter paper square saturated with concentrated CM (50-fold, concentrated by the ultrafiltration kit) was placed in the CAM surface of 6-day-old chick embryos. Three days later, the CAMs were carefully isolated and were fixed in methanol/acetone, and angiogenesis was observed under a microscope.

### 2.9. Statistical Analysis

MVD value was expressed as mean ± SD and analyzed using the two-sample *t*-test for the two groups and by analysis of variance (ANOVA) for multiple comparisons. The Mann-Whitney *U*-test was used to compare the expression levels of MTA1 and MVD values between groups. Spearman's rank correlation coefficient test was used to assess correlation between MTA1 and MVD value. Statistical analyses were carried out by SPSS 16.0 software. A value of *P* < 0.05 was accepted as statistically significant.

## 3. Results

### 3.1. Expression of MTA1 and HIF-1*α* in Residual Tumors and Tumor Periphery of Liver after TACE Treatment

**Figure 2:**
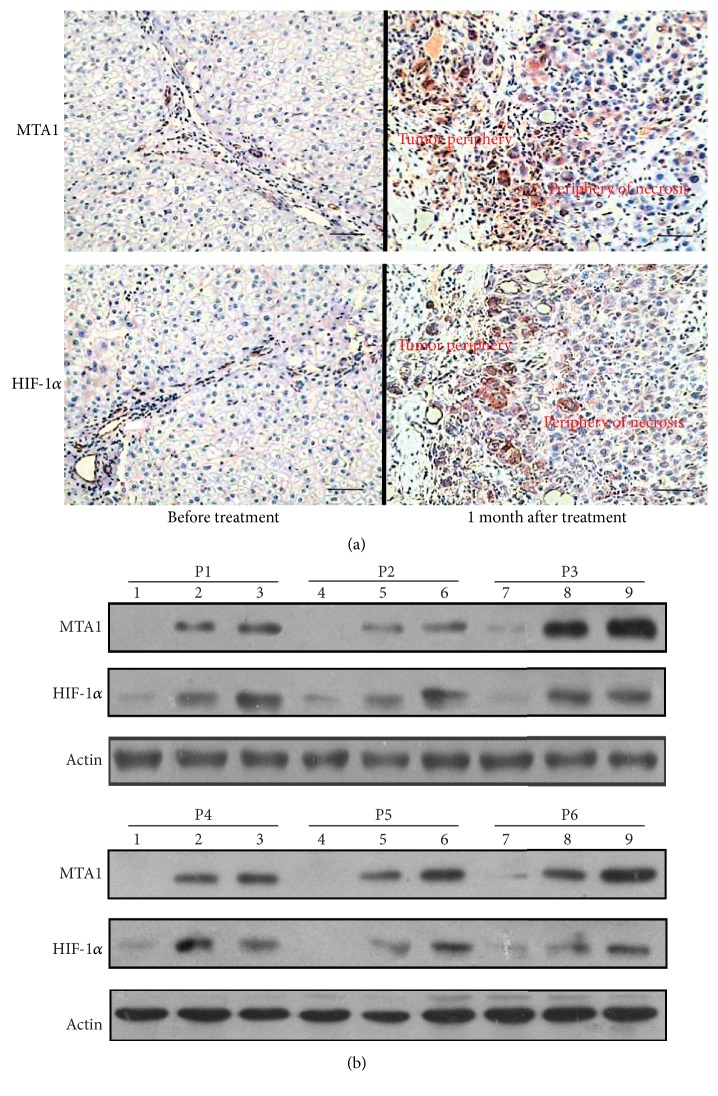
Immunohistochemistry and western blot analysis in liver tumor tissues. (a) MTA1 and HIF-1*α* staining in tumor tissues before and after TACE treatment (magnification = ×200, scale bar = 100 *μ*m). (b) Western blot results of MTA1 and HIF-1*α* in patients' samples of tumor periphery (P1 to P6). Lanes 1, 4, and 7 indicated pre-TACE treatment; Lanes 2, 5, and 8 indicated one month after TACE treatment; Lanes 3, 6, and 9 indicated two months after TACE treatment.

**Figure 3:**
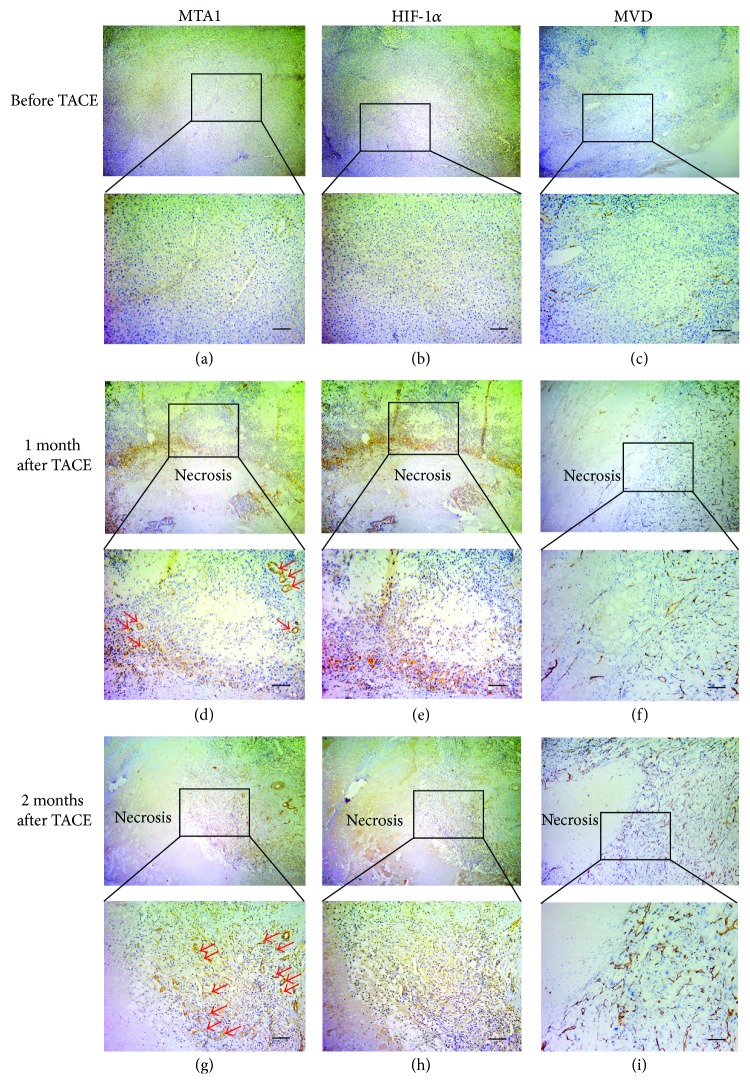
Expression of MTA1, HIF-1*α* and MVD in tumor peripheries, before TACE treatment and one month and two months after TACE treatment, respectively. Arrows indicated microvessels (magnification = ×40, and ×200, scale bar = 100 *μ*m). (a), (d), (g) MTA1 staining. (b), (e), (h) HIF-1*α* staining. (c), (f), (i) MVD (CD34 staining).

Before TACE treatment, MTA1 was stained in 34 of the 63 HCC samples in tumor tissues, but none of the surrounding liver tumor tissues were stained. Of 34 positive samples, 32 were +, 1 was ++, and 1 was +++ expression (Figure 1(b)). One month after TACE treatment, tumor tissues exhibited extensive necrosis and expression of MTA1 increased dramatically in residual tumor cells which were distributed in the border of necrotic tumor regions and the tissues of tumor periphery (Figures 2(a) and 3(d)). Moreover, upregulated expression of MTA1 sustained two months later and showed + in 25% (16/63), ++ in 30% (19/63), and +++ in 25% (16/63) versus + in 51% (32/63), ++ in 2% (1/63), and +++ in 2% (1/63) before TACE treatment, as well as + in 32% (20/63), ++ in 25% (16/63), and +++ in 21% (13/63) one month after TACE treatment (Table 2).

Positive HIF-1a staining was observed in the tumor cells after TACE, and its localization was similar to MTA1, which clustered at the periphery of substantial central necrosis and scattered at the areas near focal necrosis of tumor peripheral zone (Figures 2(a) and 3(e)). Meanwhile, results of MTA1 and HIF-1*α* expression were further validated by western blot analysis. As shown in Figure 2(b), representative samples of patients, MTA1 increased markedly in tumor periphery after TACE in Lanes 2, 5, and 8 compared with pretreated tissues in Lanes 1, 4, and 7, which further occurred in a time-dependent manner in Lanes 3, 6, and 9. Moreover, western blot analysis of HIF-1*α* was in accordance with the aforementioned changes in MTA1. Taken together, these data indicated that TACE induced the expression of MTA1 and HIF-1a.

### 3.2. Correlation between MTA1 and MVD Value

For MTA1, positivity was indicated as cytoplasmic expression. Strongly positive MTA1 staining was detected predominantly in the border of necrotic tumor regions and tumor periphery after TACE treatment, compared with most of extensively weak expression before TACE treatment (Figure 3(a)). Microvessels were heterogeneously distributed in the tumor, the most intense neovascularization was observed at the invading edge of tumor margins, and MVD value was significantly higher in TACE-treated tumor (Figures 3(f) and 3(i)). Noticeably, as shown in Figures 3(d) and 3(g), arrows indicated microvessels with MTA1 staining, whereas no HIF-1*α* staining was found in corresponding microvessels (Figures 3(e) and 3(h)).

**Figure 4:**
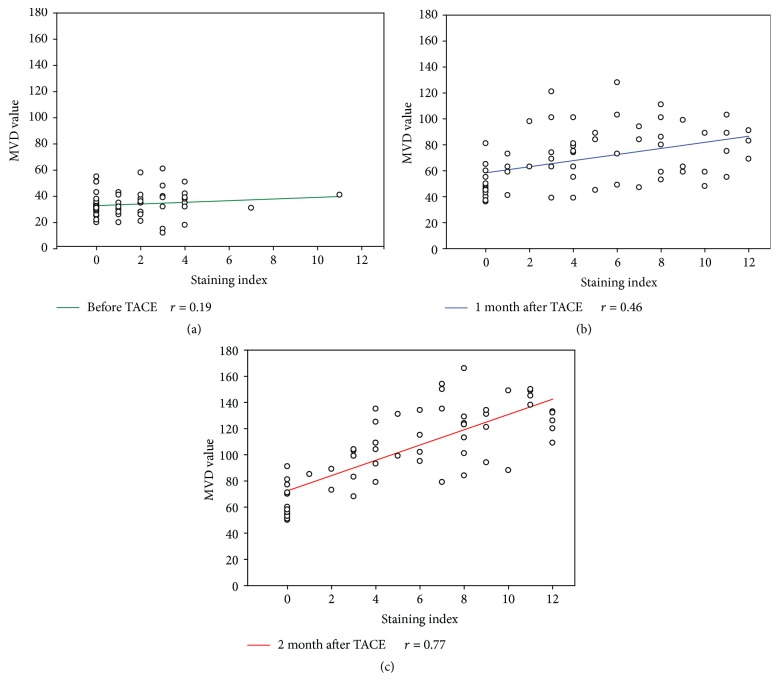
Correlation between MTA1 expression and MVD value. (a) Before TACE (*r* = 0.19, *P* > 0.1). (b) One month after TACE (*r* = 0.46, *P* < 0.01). (c) Two months after TACE (*r* = 0.77, *P* < 0.001).

Total cases of MTA1 expression increased persistently from one month to two months after TACE, which was accompanied by significantly elevated MVD value (Table 2). There was no correlation between MTA1 and MVD value before TACE treatment (Figure 4(a)), but MVD value was positively correlated with MTA1 expression after TACE treatment (Figures 4(b) and 4(c)). In general, these results demonstrated that upregulation of MTA1 enhanced vascular invasion and MTA1 played a pivotal role in angiogenesis after TACE.

### 3.3. Increased HIF-1*α* Stability and Expression by MTA1

**Figure 5:**
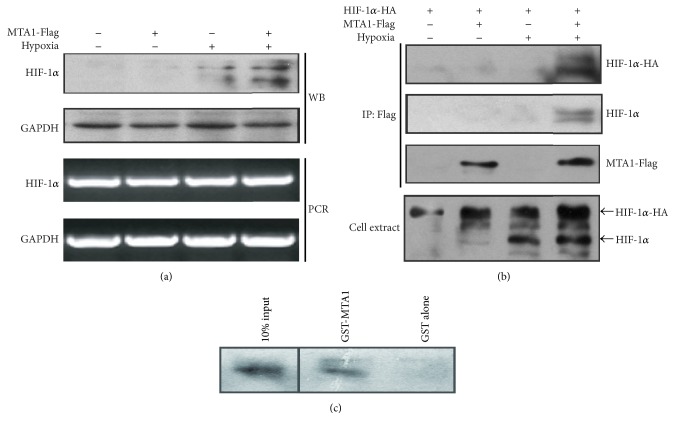
MTA1 increased HIF-1*α* stability and interacted with HIF-1*α* in vitro and in vivo. (a) HepG2 cells were stably transfected with the indicated expression vector and untreated or exposed to 1% O_2_ (hypoxia) for 4 h. (b) Mock-HepG2 and MTA1- Flag-HepG2 cells were transiently transfected with -HA or HIF-1*α*-HA as indicated, and cells were then untreated or exposed to hypoxic conditions for 4 h. (c) HIF-1*α* was translated in the presence of [^35^S]methionine and mixed with GST- or GST-MTA1-bound beads. Ten percent of the material is used as a control in this assay.

To investigate the regulation of HIF-1*α* influenced by MTA1, we overexpressed MTA1 in HepG2 cells. Consequently, the HIF-1*α* protein level was remarkably upregulated in HepG2 cells stably transfected with an MTA1 expression vector (Lane 4) (Figure 5(a)), which suggested that MTA1 was contributed to HIF-1*α* stability and expression. However, overexpression of MTA1 did not affect its mRNA level, indicating that the increase in the HIF-1*α* level by MTA1 was not due to upregulation of its transcripts, but due to posttranslational regulation instead.

### 3.4. Interaction between MTA1 and HIF-1*α*

To further explain the increased protein expression of HIF-1*α* by MTA1, we studied a possible physical interaction between HIF-1*α* and MTA1 using immunoprecipitation assay. When MTA1 transfectants were transiently transfected with HIF-1*α*-HA to enhance the expression level of HIF-1*α*, both exogenous HIF-1*α*-HA and endogenous HIF-1*α* proteins were detected in anti-Flag immunoprecipitates under hypoxic conditions (Lane 4) (Figure 5(b)), indicating binding of MTA1 with HIF-1*α* in vivo. We also studied whether MTA1 directly bound HIF-1*α* using GST-pull-down assay. As shown in Figure 5(c), translated HIF-1*α* in vitro was pulled down by GST-fused MTA1, suggesting direct interaction of HIF-1*α* with MTA1.

### 3.5. MTA1 Overexpression Enhances Angiogenesis

**Figure 6:**
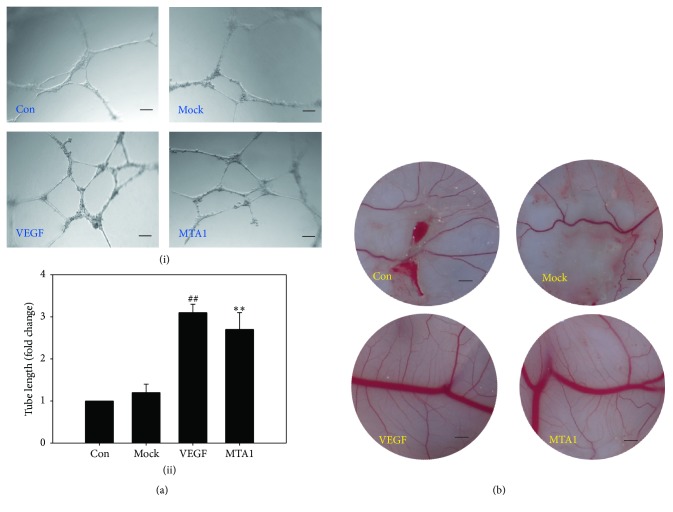
MTA1 overexpression enhanced angiogenesis. (a) CMs were collected from nontransfected (Con), mock-transfected (Mock), or MTA1-transfected HepG2 cells (MTA1). HUVECs on matrigel were grown in the CM for 24 h. VEGF (10 ng/mL) was employed as a positive control. (i) Representative images showing tube formation (magnification = ×40, scale bar = 100 *μ*m). (ii) Tube length was quantified and expressed as the means ± SD (*n* = 5). ^##^*P* < 0.01 (VEGF versus Con); ^*∗∗*^*P* < 0.01 (MTA1 versus Con). (b) The chick embryos were treated with CMs collected from nontransfected (Con), mock-transfected (Mock), or MTA1-transfected HepG2 cells (MTA1). VEGF (30 ng/mL) was employed as a positive control (magnification = ×20, scale bar = 200 *μ*m).

In order to investigate the biological consequences of HIF-1*α* stabilization by MTA1, we conducted tube formation in vitro and CAM assays in vivo. The tube formation assay showed that CM collected from MTA1 transfectants with overexpression induced an increased formation of the organized network of tubular structures on matrigel compared with control CM or mock CM (Figure 6(a)). The CAM assay also demonstrated that the concentrated CM (50-fold) collected from MTA1 transfectants had a significantly higher angiogenic activity with more new blood vessels and larger lumen of vessels than that collected from control or mock transfectants (Figure 6(b)). These data confirmed that the increased stability of HIF-1*α* by MTA1 led to the increased angiogenesis.

## 4. Discussion

Nowadays, application of TACE is increasing to treat HCC. However, TACE treatment tends to result in angiogenesis, leading to local recurrence and metastases. Antiangiogenic strategy, such as bevacizumab therapy targeting the vascular endothelial growth factor (VEGF) pathway, is often ineffective [20], implying that other factors are also important in tumor angiogenesis, including the upstream of angiogenesis. Therefore, elucidation of TACE-induced neovascularization is a significant concern.

In the previous study, Hamatsu et al. found that high mRNA expression of the MTA1 gene in HCC was recognized in 42% samples, as compared to the paired nontumor tissues [21]. The phenomenon was further confirmed in another investigation; Moon et al. examined MTA1 protein expression in resected human HCC specimens; the predominantly positive expression was up to 69% [22]. Our results were consistent with the above-mentioned studies; MTA1 was stained in 34 of 63 HCC patients before treatment, which accounted for 54%. Noticeably, expression of MTA1 increased, especially in tumor periphery with dramatical upregulation after TACE. A rim of viable tumor cells is observed in tumor periphery which is close to the normal surrounding tissue, due to uncompleted blockade of the arterial blood supply and endurance in hypoxia and ischemia. The aforementioned expression of MTA1 in tumor periphery is significantly activated after TACE, and thus upregulation of MTA1 is considered to the new finding of the response to hypoxia. Proliferation in periphery of residual cells is the main cause to recurrence, and residual cells need formation of new peritumoral blood vessels for oxygen and nutrition supply to survive. In the present study, strongly positive MTA1 staining was found in microvessels of the tumor border (Figures 4(b) and 4(c)), and Spearman rank correlation coefficient test further showed positive correlation between MTA1 and MVD value after TACE (Figures 3(d) and 3(g)), indicating that MTA1 was contributed to the neovascularization of residual tumors.

Activation of HIF-1*α* under hypoxic conditions is instantaneous [23], which allows cancer cells to adapt to hypoxic environments by controlling the signal transduction cascades of plenty of downstream effectors, including VEGF, glycolytic enzymes, glucose transporters, and erythropoietin related to angiogenesis, metabolism, and proliferation [24]. It has been recently reported that MTA1 enhances the transcriptional activity of HIF-1*α* in human breast cancer cells [25], but the relationship between MTA1 and HIF-1*α* in HCC is unknown. Here in the study, expression of HIF-1*α* increased significantly in periphery of necrotic tumor regions and tumor periphery one month after TACE and positive expression sustained two months later, which was in accordance with the pattern of alterations in MTA1. Namely, activation of HIF-1*α* was accompanied by MTA1 upregulation. Therefore, MTA1 was involved in the effect on HIF-1*α* and played the role in upstream signaling pathway of angiogenesis.

In order to further elucidate mechanism of interaction between MTA1 and HIF-1*α*, hepatocellular carcinoma cell line HepG2 was employed. We found the expression of HIF-1*α* protein was enhanced in stable MTA1 transfectants of HepG2 cells under hypoxic conditions, but the expression of its mRNA was not changed (Figure 5(a)). It was suggested that MTA1 increased the stability of HIF-1*α*. We further demonstrated that MTA1 interacted with HIF-1*α* both in vitro and in vivo (Figures 5(b) and 5(c)); MTA1 was the proangiogenic cytokine that bound HIF-1*α* through direct interaction, leading to stabilization of HIF-1*α*. Moreover, results of tube formation assay and CAM assay confirmed the effect of MTA1 on proangiogenic activity (Figures 6(a) and 6(b)).

## 5. Conclusions

Our findings provided strong evidence that MTA1 was an active angiogenic regulator. The mechanism underlying was related to direct binding of MTA1 with HIF-1*α* in hypoxia, leading to increased stability of HIF-1*α* and proangiogenic capability. The change of MTA1 is the important characteristic in TACE-treated patients, and MTA1 expression level might be a prognostic indicator for TACE. Recently, natural agents targeting MTA1 or MTA1/NuRD complex have exhibited good curative efficacy in tumors [26–28]. Therefore, interruption of MTA1-mediated angiogenesis is a considerable strategy for improving therapeutic effects of TACE treatment on HCC.

## Figures and Tables

**Table 1:** Characteristics of HCC patients receiving TACE treatment.

Features	Patients
Gender (*n*)	
Male	39
Female	24

Age (year)	
Average	51.5
Range	39–71

Location (*n*)	
Right hepatic lobe	36
Left hepatic lobe	27

Child-Pugh classification (*n*)	
Grade A	34
Grade B	29

Tumor size (mm)	
Average	63.1
Range	38.2–129.1

**Table 2:** Comparison of MTA1 expression and MVD value, before and after TACE treatment.

Time	MTA1				MVD Per 200x field (means ± SD)
	−	+	++	+++	
Before TACE	29/63	32/63	1/63	1/63	33.8 ± 9.6
1 month after TACE	14/63	20/63	16/63	13/63	68.6 ± 22.7^*∗∗*^
2 months after TACE	12/63	16/63	19/63	16/63	112.1 ± 30.6^*∗∗∗*^

^*∗∗*^ *P* < 0.01 (MVD value 1 month after TACE versus MVD value before TACE),

^*∗∗∗*^ *P* < 0.001 (MVD value 2 months after TACE versus MVD value before TACE).
